# Endogenous fungal endophthalmitis caused by *Cladophialophora devriesii*: report of a case and literature review

**DOI:** 10.1186/s12348-024-00408-y

**Published:** 2024-06-05

**Authors:** Jørgen Krohn, Øystein A. Power, Haima Mylvaganam, Andreas J. Askim, Jarle B. Arnes, Bjørn Blomberg

**Affiliations:** 1 Department of Clinical Medicine, Section of Ophthalmology, University of Bergen, Bergen, Norway; 2 Department of Ophthalmology, Haukeland University Hospital, Bergen, Norway; 3 Department of Medicine, Haukeland University Hospital, Bergen, Norway; 4 Department of Microbiology, Haukeland University Hospital, Bergen, Norway; 5 Department of Pathology, Haukeland University Hospital, Bergen, Norway; 6 Department of Clinical Science, Infectious Diseases Section, University of Bergen, Bergen, Norway

**Keywords:** *Cladophialophora devriesii*, *Cladosporium* species, Dematiaceous fungus, DNA sequence analysis, Endogenous endophthalmitis, Fungal endophthalmitis, Phaeohyphomycosis, Triazole derivatives

## Abstract

**Purpose:**

To report a case of endogenous endophthalmitis caused by the dematiaceous fungus *Cladophialophora devriesii*.

**Methods:**

Observational case report and literature review.

**Case presentation:**

A 73-year-old female with a history of chronic obstructive pulmonary disease presented with a red and painful left eye. Examination revealed anterior segment inflammation and vitritis, indicative of endophthalmitis. She underwent core vitrectomy and intravitreal injection of vancomycin and amphotericin B. The vitreous sample showed inflammatory cells and fungal hyphae, and systemic amphotericin B and itraconazole were commenced for fungal endophthalmitis. Targeted amplification of the sample for bacterial DNA (V2-V3 region of 16 S rDNA) was negative, but fungal DNA targets (ITS1 and ITS2) were present, and their sequences were consistent with *Cladophialophora devriesii*. Phenotypic characterisation and sequencing of ITS1 and ITS2, carried out on cultured fungus from the sample, also revealed *Cladophialophora devriesii*. She received repeated intravitreal injections of voriconazole, and based on the antifungal susceptibility results, her systemic medication was changed to posaconazole. After 12 months, the eye showed no signs of inflammation, and posaconazole therapy was discontinued. After 3 months without antifungal medication, the inflammation recurred, and she was restarted on antifungal therapy for an additional 20 months. Another recurrence occurred 3 months after discontinuation of treatment, and a repeat vitreous sample confirmed the presence of *Cladophialophora devriesii*. She was started on isavuconazole, but developed seclusio pupillae and painful secondary glaucoma. Due to the duration and severity of the infection, the eye was enucleated. Histopathology revealed persistent fungal elements at the ciliary processes and the posterior lens surface.

**Conclusions:**

This second reported case of endogenous endophthalmitis caused by *Cladophialophora devriesii* illustrates the role of vitreous sampling and molecular methods in diagnosis and treatment of fungal endophthalmitis. Despite early diagnosis and prolonged local and systemic antifungal therapy, it was not possible to achieve long-term control of the fungal infection.

## Introduction

Dematiaceous or black fungi are a heterogeneous group of fungi widely distributed in soil and decomposing plant material, mainly in tropical and subtropical regions [1]. They are characterised by the presence of melanin in their cell walls, leading to brown hyphae and conidia that form characteristic dark-coloured colonies [2]. Dematiaceous fungi can cause a variety of human infections, known as chromoblastomycosis and phaeohyphomycosis, and their ability to produce melanin is considered as an important virulence factor [3, 4]. Chromoblastomycosis, also called chromomycosis, is a chronic granulomatous infection of the skin and subcutis where, histopathologically, so-called Medlar bodies or sclerotic bodies are present in the affected tissue [5]. Phaeohyphomycosis can occur in both immunocompromised and immunocompetent individuals and lead to cutaneous, soft tissue, respiratory tract, and central nervous system (CNS) infections [4].

Most cases of ocular phaeohyphomycosis are exogenous and acquired through contaminated corneal or conjunctival injuries or following ocular surgery [6]. The most common dematiaceous fungal eye infection is keratitis, and its incidence appears to increase [7]. Endophthalmitis following disseminated fungaemia typically occurs in individuals with recent hospitalisation, major surgeries or underlying diabetes, malignancy or other systemic conditions affecting their immune system [8, 9]. Fungal endophthalmitis is usually endogenous in nature and often caused by *Candida* or *Aspergillus* species [9–11]. Only a few cases of endogenous endophthalmitis caused by dematiaceous fungi have been reported in the literature, and the prognosis is generally poor [12].

*Cladophialophora* (formerly known as *Cladosporium*) is a genus of dematiaceous hyphomycetes in the family *Herpotrichiellaceae*, which includes several pathogenic species [13, 14]. Herein, we present a challenging case of endogenous endophthalmitis caused by *Cladophialophora devriesii* and provide a brief review of the literature.

### Case presentation

A 73-year-old Caucasian female had noticed blurred vision and floaters in her left eye for about five months. One week prior to admission, she experienced increasing redness and pain in the same eye. There was no recent history of trauma or foreign travel. Her past medical history included tachycardia-bradycardia syndrome treated with pacemaker implantation 10 years earlier, and recurrent respiratory tract infections and chronic obstructive pulmonary disease (COPD) over the last 20 years. Treatment comprised of inhaled salmeterol/fluticasone propionate and short courses of prednisolone once or twice per year, typically administered over 4 weeks, starting at 20–30 mg/day and rapidly tapered to 5 mg/day.

**Fig. 1:**
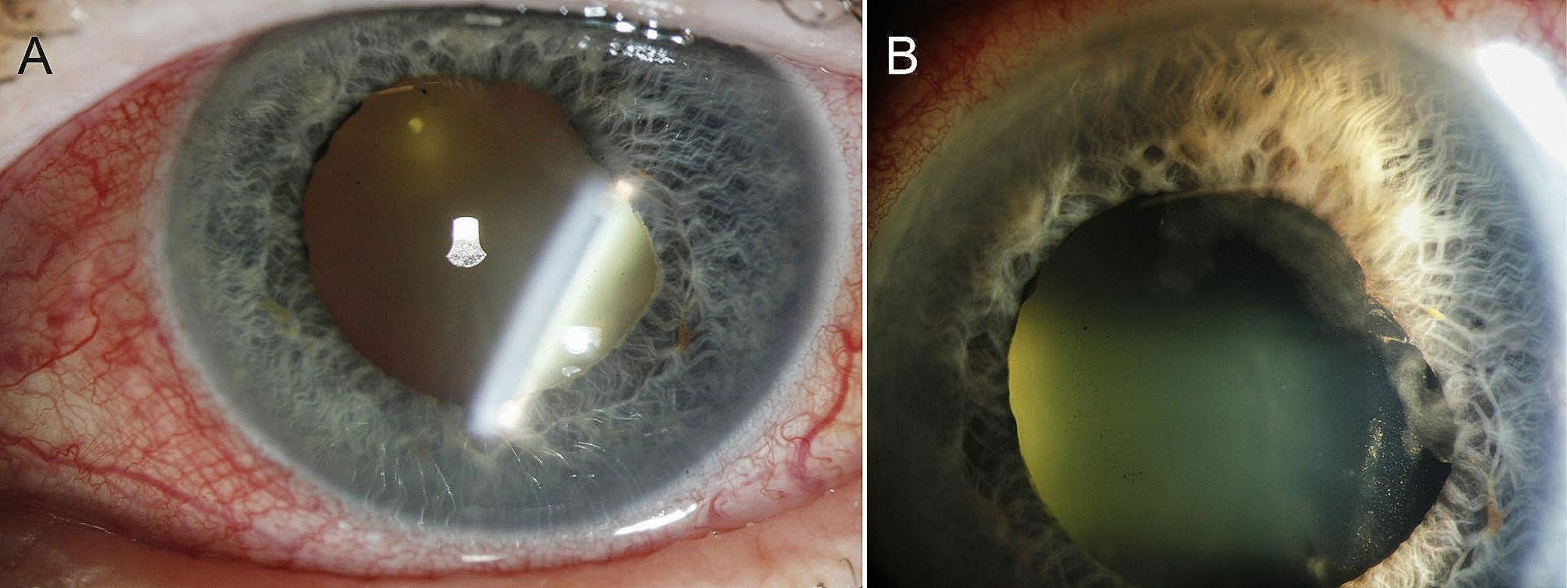
Anterior segment photographs of the left eye at presentation. (**A**) Note the marked injection of the eye and the mild nuclear cataract. (**B**) Closer view of the anterior chamber shows some fibrin and posterior synechia

At initial examination, the patient’s best corrected visual acuity (BCVA) was 6/7.5 in the right eye and 6/15 in the left eye. Intraocular pressure (IOP) was 10 mmHg and 5 mmHg, respectively. Anterior segment examination of the left eye revealed mixed conjunctival and ciliary injection, fine inferior keratic precipitates, anterior chamber cells and flare, fibrin formation with a small posterior synechia, and mild nuclear cataract (Fig. 1A, B). In the posterior segment, there was moderate vitritis with yellow-white condensations inferiorly. Fundoscopy through hazy media did not show any chorioretinal abnormalities. Examination of her right eye was unremarkable except for minimal nuclear cataract and a few macular drusen.

Complete blood count and biochemistry results were normal, apart from slightly elevated C-reactive protein level of 13 mg/L (reference < 5 mg/L). Urinalysis and culture performed shortly prior to admission were negative. Computed tomography (CT) of the head and brain showed normal sinuses and no evidence of fungal infection or malignancy. A comprehensive evaluation by an ear, nose and throat specialist, including rhinoscopy, revealed no evidence of sinusitis or upper respiratory tract infection. On chest CT and X-ray, bilateral infiltrates, basal condensations, and bronchiectasis consistent with pneumonia and COPD were observed. Whole-body positron emission tomography (PET)/CT showed a few pulmonary foci suggestive of active pneumonia. Transthoracic echocardiography demonstrated normal valves without vegetations. Repeated blood cultures were all negative. Bronchoalveolar lavage fluid showed growth of *Haemophilus influenzae*, but was negative by both microscopy and culture for fungi and mycobacteria.

**Fig. 2:**
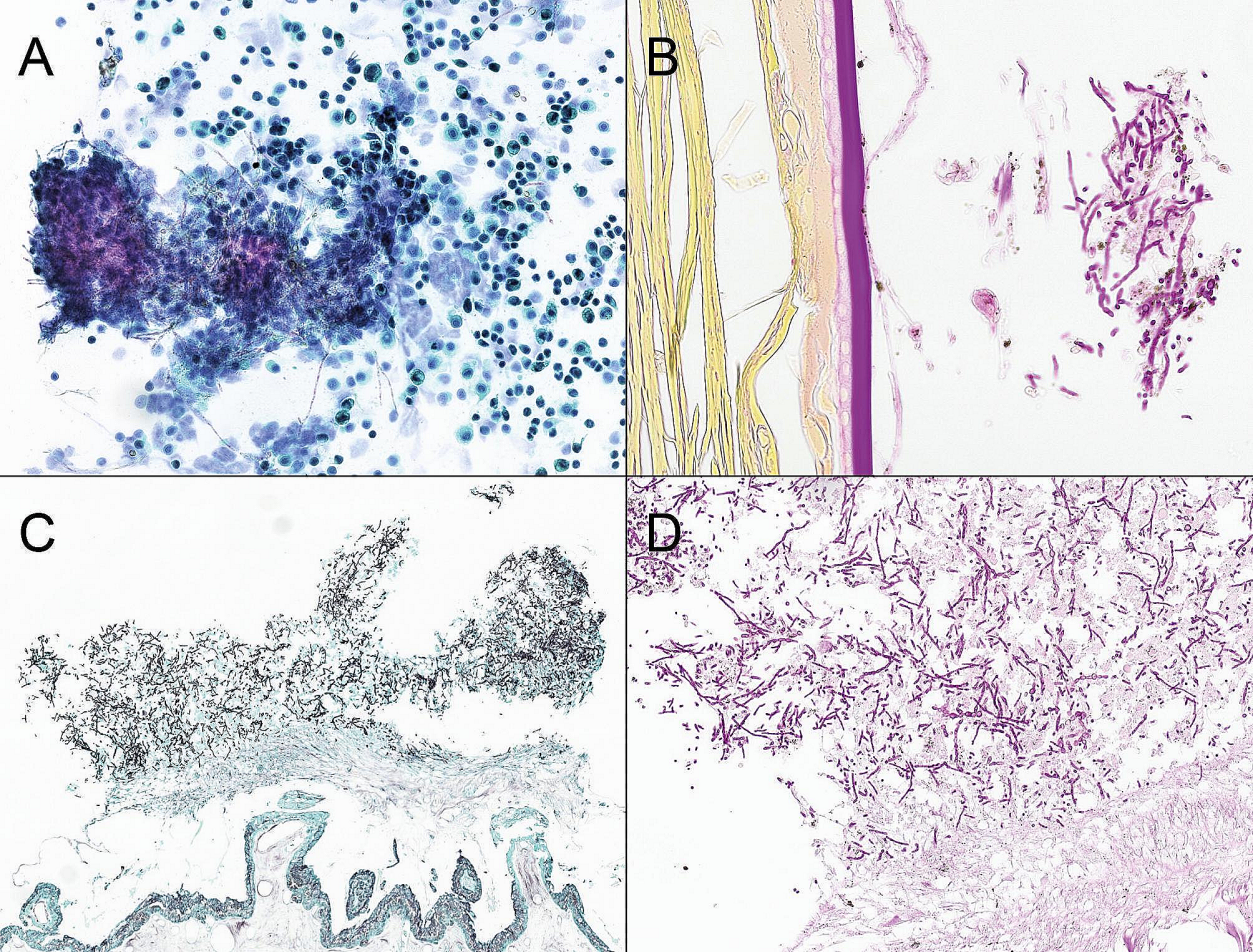
(**A**) Cytopathological examination of the vitreous specimen reveals fungal hyphae together with inflammatory and degenerated cells (Papanicolaou stain). (**B**) Histopathology of the enucleated eye reveals fungal hyphae located at the posterior lens capsule. The lens fibres are seen to the left of the vertically oriented posterior lens capsule (Periodic acid-Schiff stain). (**C**) Clusters of hyphae and conidia surrounded by inflammatory cells and cellular debris close to the ciliary processes, which are seen in the lower part of the image (Grocott methenamine silver stain). (**D**) At higher magnification, further details are revealed (Periodic acid-Schiff stain)

**Fig. 3:**
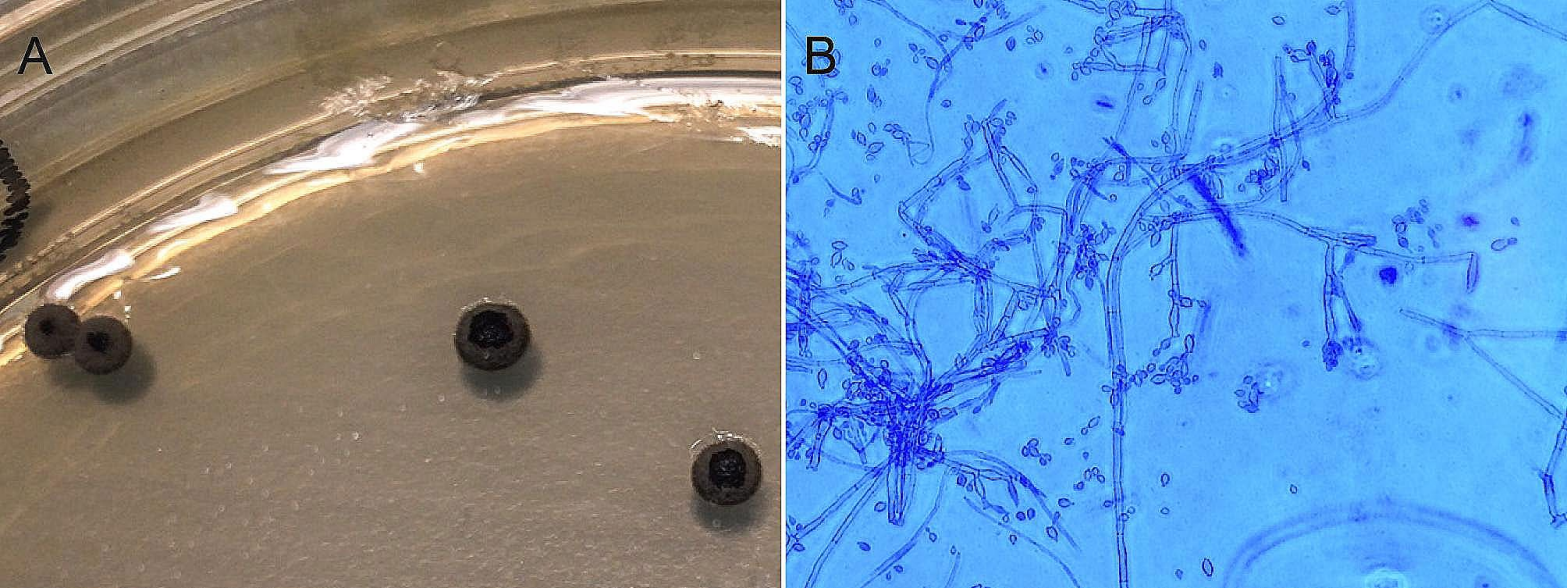
(**A**) Culture on Sabouraud dextrose agar shows growth of small, grey-black colonies after one week of incubation. (**B**) Lactophenol cotton blue preparation of the colony material shows septate hyphae with solitary and short chains of ellipsoidal to fusiform conidia consistent with *Cladophialophora* species

**Fig. 4:**
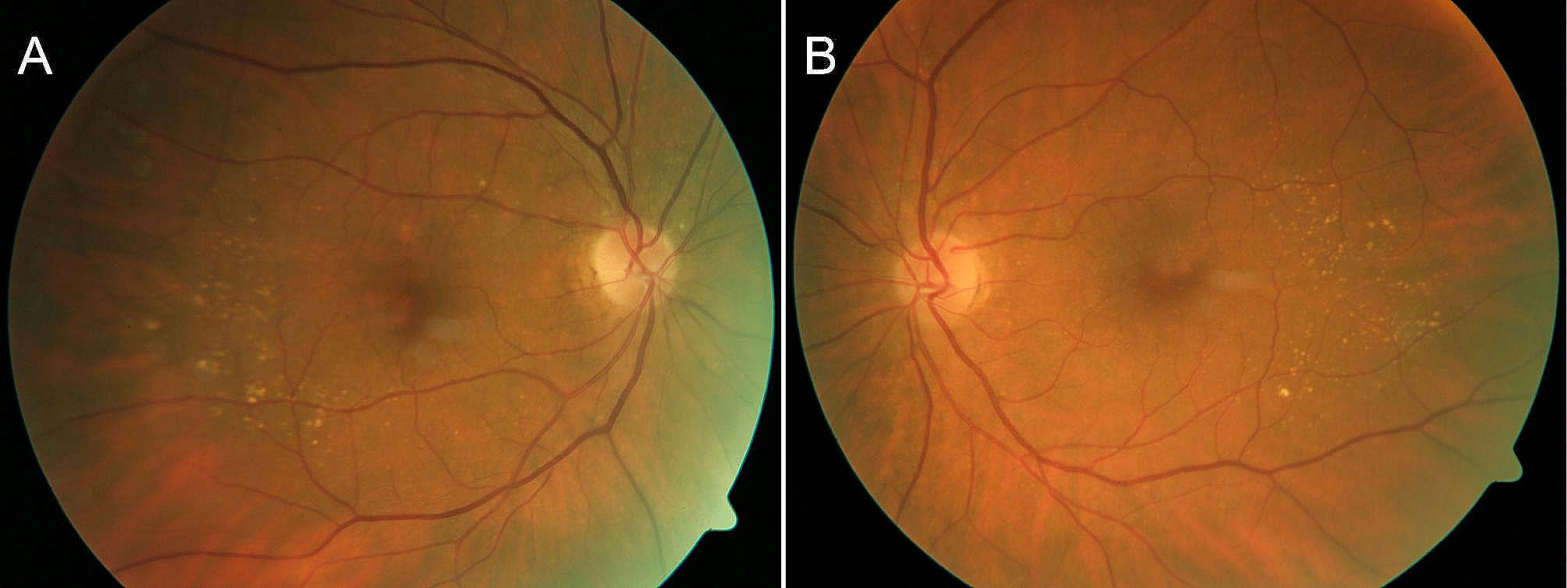
Fundus photographs of (**A**) the right eye and (**B**) the left eye taken during the course of treatment. Small drusen temporal to the macula are seen in both eyes. There are no inflammatory chorioretinal lesions or infiltrates in the left (endophthalmitis) eye

Three days after admission, BCVA in her left eye was reduced to 6/30, and a core vitrectomy, followed by intravitreal injection of vancomycin 1 mg and amphotericin B 5 µg, was performed. No inflammatory lesions or thickening of the anterior choroid and ciliary body were observed during the vitrectomy. Microscopic examination of the vitreous showed inflammatory cells including macrophages and granulocytes, and the presence of fungal hyphae (Fig. 2A). Atypical lymphoid cells were not observed. Targeted amplification by polymerase chain reaction of the V2-V3 region of bacterial 16 S ribosomal deoxyribonucleic acid (16 S rDNA) was negative. Targeted amplification of fungal DNA targets, namely, internal transcribed spacers 1 and 2 (ITS1 and ITS2) and D1-D2 region of 28 S rDNA, were present. The sequence of D1-D2 region of 28 S rDNA could not differentiate between *Fonsecaea* and *Cladophialophora* species. However, sequences of ITS1 and ITS2 identified the microbe correctly to its species level as *Cladophialophora devriesii*. She was started on intravenous liposomal amphotericin B 150 mg (3 mg/kg) once daily and oral itraconazole 200 mg twice daily. She also received oral cotrimoxazole for a probable *Haemophilus influenzae* pulmonary infection. Culture of the vitreous sample demonstrated grey-black colonies after one week of incubation and their morphology and microscopic findings were consistent with *Cladophialophora* species (Fig. 3A, B). The ITS2 sequence obtained from the colonies was identical to that obtained from the sample. Then, the first intravitreal injection of 75 µg voriconazole was given. The eye gradually became less inflamed and BCVA improved to 6/12. A second intravitreal injection of voriconazole was given 10 days later. After two weeks on systemic amphotericin B and itraconazole treatment, her antifungal medication was changed to oral voriconazole 200 twice daily. During the following days, she complained of nausea, dizziness, and fatigue and appeared slightly confused. Brain CT was normal and transoesophageal echocardiography showed normal valves. Voriconazole-related side effects were suspected, and her medication was switched back to oral itraconazole 200 mg twice daily. At follow-up examination four weeks later, the eye showed no signs of inflammation, but progression of nuclear cataract was observed. The results of antifungal susceptibility testing by E-test were available from our reference laboratory about six weeks after the vitreous sampling and showed that posaconazole had the lowest minimum inhibitory concentration (MIC) value (0.008 mg/L) (Table 1). Her antifungal therapy was switched to oral posaconazole 300 mg daily. Despite troublesome side effects, such as myalgia, dry cough, and subjective hearing impairment, she continued this treatment for a total of 12 months. Whole-body PET/CT showed no signs of fungal infections, including pneumonia, at this stage. Examination of the left eye revealed nuclear cataract, otherwise both eyes were quiet without injection, anterior chamber cells, and vitritis (Fig. 4A, B).

**Table 1:** Minimum inhibitory concentration values of various antifungals against *Cladophialophora devriesii* isolates obtained from vitreous samples taken at admission (Isolate 1) and more than 3 years later (Isolate 2)

Antifungal agent	Isolate 1		Isolate 2
	E-test MIC (mg/L)	Broth dilution MIC (mg/L)	E-test MIC (mg/L)
Amphotericin B	32	1	4
Fluconazole	16	ND	ND
Isavuconazole	0.016	0.125	0.004
Itraconazole	ND	0.064	0.032
Posaconazole	0.008	0.32	0.002
Voriconazole	0.016	0.25	0.004
Anidulafungin	1	ND	0.008
Caspofungin	0.5	ND	0.125
Micafungin	0.5	ND	0.25

MIC minimum inhibitory concentration, ND not determined

**Fig. 5:**
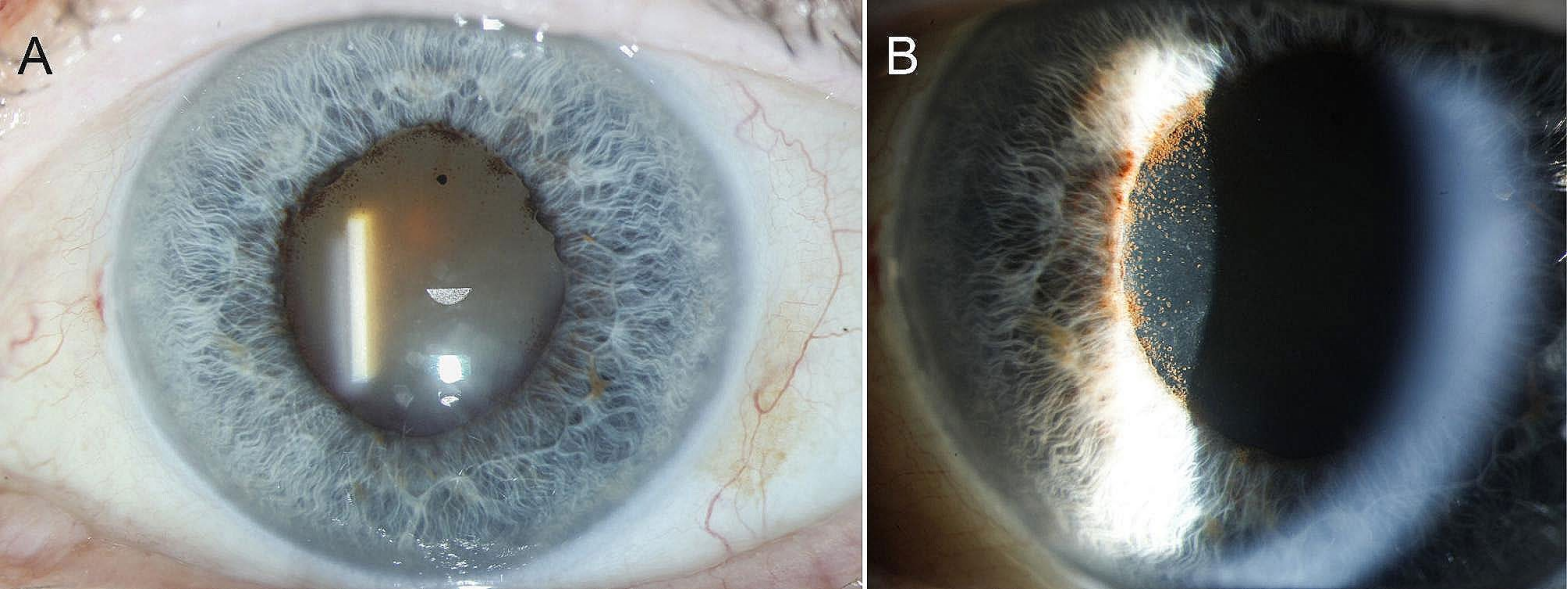
Anterior segment photographs of the left eye taken at recurrence more than three years after initial presentation. (**A**) Note the lack of injection, the secluded pupil, and the dense nuclear cataract. (**B**) Closer view of the anterior chamber shows extensive posterior synechiae and pigment deposits on the lens surface

After three months without any antifungal medication, the patient noted increasing redness and pain in the left eye. Her BCVA was 6/38 and IOP was 4 mmHg. The eye was injected with keratic precipitates, anterior chamber cells and flare, extensive posterior synechiae, and marked nuclear cataract precluding clear fundus viewing. Repeated haematological, biochemical, and radiological investigations were all negative. She underwent a vitreous tap with intravitreal injection of 5 µg amphotericin B and was started on oral posaconazole 300 mg daily, as well as topical steroids and cyclopentolate. Intravitreal injection of amphotericin B was repeated after one week, followed by intravitreal injection of 100 µg voriconazole. Microscopy, cultures, and sequencing results of the vitreous sample were negative for fungi and bacteria. Redetermination of MIC by broth dilution method was then performed at the Statens Serum Institut in Denmark, using the fungal isolate cultured from the initial sample (taken 16 months earlier), and markedly different results were obtained (Table 1). Posaconazole was replaced by oral isavuconazole 200 mg daily, but after about six weeks, her medication had to be switched back to posaconazole 300 mg daily due to limited drug availability. After a further 18 months on oral posaconazole, the eye showed no signs of active inflammation, and once again an attempt to discontinue her antifungal medication was made. At follow-up examination three months later, the left eye was white, but a significant increase in anterior chamber cells and flare was observed. There was also a marked progression of the nuclear cataract and almost complete posterior synechiae (Fig. 5A, B). Visual acuity was reduced to finger counting at 2 m and IOP was 14 mmHg. Another vitreous sample, taken more than three years after the initial identification of *Cladophialophora devriesii*, revealed growth of the same fungus with identical ITS2 sequence. MIC values by E-test of this isolate, performed at our reference laboratory, were slightly different from the corresponding values of the initial isolate, but did not indicate development of resistance to triazole derivatives (Table 1). She was started on isavuconazole 200 mg daily in addition to topical steroids and cyclopentolate. During the following two weeks, the eye remained inflamed and she developed seclusio pupillae, which subsequently led to iris bombé and painful secondary glaucoma with an IOP of 45 mmHg. Due to the duration and severity of the infection, the eye was finally enucleated. Histopathologic examination of the enucleated eye revealed the presence of fungal hyphae and conidia close to the ciliary processes and the posterior lens surface (Fig. 2B–D). During three years of follow-up, the patient has been in good condition without any signs of systemic fungal disease.

## Discussion

*Cladophialophora* are dematiaceous hyphomycetes or conidial moulds that are ubiquitous and distributed worldwide in soil and decaying organic material [13, 14]. *Cladophialophora carrionii* is one of the most frequent species implicated in human diseases, particularly chromoblastomycosis [15, 16]. The most common species causing CNS phaeohyphomycosis is *Cladophialophora bantiana*, which has a marked tendency to form cerebral abscesses due to its neurotrophic properties [17, 18]. *Cladophialophora devriesii* is an uncommon human pathogen that has been known to cause subcutaneous and fatal disseminated phaeohyphomycosis [14, 19–21].

Dematiaceous fungi are rare agents of endophthalmitis, and among them, only a few cases of endophthalmitis caused by various *Cladophialophora* species have been reported. Table 2 summarises previously reported cases of fungal endophthalmitis caused by *Cladophialophora* species, in addition to the present patient [22, 23]. To our knowledge, there is only one previously reported case of endophthalmitis caused by *Cladophialophora devriesii*, which like our case was of endogenous origin [22]. In the table, we have chosen to also include previously published cases of endophthalmitis caused by unidentified species of the dematiaceous genus *Cladosporium* [24–28]. This was done because many *Cladophialophora* species are still referred to by their old nomenclature *Cladosporium* or they might have been incorrectly identified since the two genera share several morphological features [29, 30].

**Table 2:** Summary of reports of fungal endophthalmitis caused by *Cladophialophora* and unidentified *Cladosporium* species

Authors, year, [Ref]	Country	Age, sex	Eye	Predisposing factors	Type of endophthalmitis	Systemic infectious foci	Fungal pathogen	Treatment	Follow-up (months)	Outcome
Raspiller et al., 1988 [24]	France	NAv, male	OS	Perforating corneal injury	Posttraumatic exogenous	NApp	*Cladosporium sp.*	Subconjunctival amphotericin B. Systemic ketoconazole, miconazole, and nystatin	0	VA: LP. Died of probable Reye’s syndrome during treatment
Schiedler et al., 2004 [22]	USA	69, male	OS	Intravenous catheter, lymphoma, heart transplant, immunosuppressive therapy	Endogenous	Endocarditis, vocal cord infection	*Cladophialophora devriesii*	Vitrectomy. Intravitreal amphotericin B. Systemic amphotericin B and fluconazole	10	VA: 20/20. Died of fungaemia 10 months after treatment
Wu et al., 2011 [25]	Taiwan	29, female	OS	Postpartum	Endogenous	Unknown	*Cladosporium sp.* and *Candida albicans*	Vitrectomy. Intravitreal amphotericin B, fluconazole, and voriconazole. Systemic voriconazole	10	VA: 20/25. Resolved
Doshi et al., 2019 [23]	India	62, male	OD	Diabetes mellitus, cataract surgery	Postoperative exogenous	NApp	*Cladophialophora sp.*	Cryotherapy. Intravitreal amphotericin B and voriconazole	6	VA: 20/30. Resolved
Dimacali & Lim Bon Siong, 2020 [26]	Philippines	NAv	NAv	Keratoplasty	Postoperative exogenous	NApp	*Cladosporium sp.*	NAv	NAv	NAv
Abd Hamid et al., 2022 [27]	Malaysia	52, male	OD	Rectal carcinoma, gastrointestinal surgery, chemoradiotherapy	Endogenous	Unknown	*Cladosporium sp.*	Vitrectomy. Intravitreal amphotericin B and voriconazole. Systemic fluconazole	1	VA: CF. Foveal scar with epiretinal membrane
Kuo et al., 2022 [28]	Taiwan	NAv	NAv	NAv	Endogenous	NAv	*Cladosporium sp.*	NAv	NAv	NAv
Current report	Norway	73, female	OS	Chronic obstructive pulmonary disease, pneumonia, intermittent corticosteroid therapy	Endogenous	Presumably pulmonary	*Cladophialophora devriesii*	Vitrectomy. Intravitreal amphotericin B and voriconazole. Systemic amphotericin B, itraconazole, voriconazole, posaconazole, and isavuconazole	80	Seclusio pupillae and secondary glaucoma. Enucleation

CF counting fingers, LP light perception, NApp not applicable, NAv not available, OD right eye, OS left eye, Ref reference, sp. species, VA visual acuity

Most cases of endogenous fungal endophthalmitis develop in immunocompromised patients and rarely in healthy individuals without any known risk factors [11]. Our patient had COPD, was on continuous treatment with inhalation steroids, and received short courses of oral steroids at irregular intervals, all of which may contribute to mild immunosuppression. Beyond this, she had no known immune dysfunction. She denied any trauma to the eye region and had no history of foreign travel for many years preceding the fungal infection. Although the source of the endogenous *Cladophialophora* endophthalmitis in our patient is unknown, we assume it could be associated with her COPD, which is known to increase the susceptibility to invasive fungal infections [31]. Also, the negative blood cultures and the negative cultures and cytology of the bronchoalveolar lavage samples do not rule out haematogenous spread from an unrecognised pulmonary focus, as such tests are frequently negative even in active pulmonary fungal diseases [32, 33]. We considered the possibility that the fungus might have been introduced during the implantation of the pacemaker. However, this was deemed unlikely since it was a relatively minor procedure that predated the infection by a decade, and the patient had no evidence of pacemaker-associated infection at any time point.

Infections caused by slow growing fungi can be indolent and slowly progressive, causing significant diagnostic and therapeutic delay [12, 34]. In our case, vague symptoms suggestive of infection were present for several months until increased redness and pain led to hospitalisation. In contrast to what is usually found in endogenous fungal endophthalmitis, there was no evidence of chorioretinal lesions or infiltrates [34]. As endogenous fungal endophthalmitis usually begins with haematogenous seeding of the choroid with subsequent vitreous involvement, it is tempting to speculate that the primary entrance point into the eye was located more anteriorly in the uveal tract (i.e., the ciliary body or iris) [9]. This corresponds to the histological findings of fungal elements close to the ciliary body epithelium and the posterior lens surface. The anteriorly located focus of infection may also have contributed to the relatively rapid and asymmetric nuclear cataract formation [35]. Owing to the slow and unilateral inflammation with vitreous involvement, fungal endophthalmitis was initially suspected. Vitreoretinal lymphoma was considered as a possible differential diagnosis, but CT of the brain showed no evidence of CNS lymphoma and cytological examination of the vitreous did not reveal atypical lymphoid cells. Although immune-related uveitis could not be excluded, both local and systemic steroid therapy were avoided until the cytology and culture results were known. Cytology of the vitreous sample revealed fungal hyphae, and sequencing of the ITS2 region of fungal DNA from the sample showed a high degree of homology with *Cladophialophora* species. After about two weeks, the fungus isolated and cultured from the sample was identified as *Cladophialophora devriesii*. In our case, molecular biological methods allowed for early identification and targeted antifungal therapy pending culture results. Successful DNA-based identification is dependent on an adequate sample size, selection of good gene targets suitable for correct identification to the species level, bidirectional DNA sequencing to ensure the quality of the sequence, and the availability of comprehensive and reliable databases for comparison. Our experience with *Cladophialophora devriesii* showed that the D1-D2 region of the 28 S rDNA is inferior to ITS1 and ITS2 for correct identification.

Determination of MIC values for moulds other than *Aspergillus* species provides only a guide to treatment, and the method is not generally recommended as the lack of clinical breakpoints and epidemiological cut-off values makes the results difficult to interpret [36, 37]. Furthermore, the values show considerable methodological variations, as illustrated by the different MIC values of the same culture obtained first by the E-test and later by the broth dilution method (Table 1). It is noteworthy that the European Committee on Antimicrobial Susceptibility Testing recommends broth dilution method for moulds, but interpretative guidelines are not yet available for non-*Aspergillus* moulds [38]. In addition, a correlation between in vitro susceptibility results and clinical response has been difficult to demonstrate [36]. Correct identification to the species level and relevant reports on treatment outcomes are crucial for choosing appropriate antifungal therapy in a clinical setting. Several triazole derivatives, including itraconazole, posaconazole, voriconazole, and isavuconazole have been reported to be clinically effective against *Cladophialophora* species and other dematiaceous fungi [39, 40]. Over the course of our patient’s treatment, they were all used separately for different durations due to various side effects and limited drug availability.

Following core vitrectomy and one year of intravitreal and systemic triazole therapy, her endophthalmitis seemed to be under control, but recurred after three months without medication. This is consistent with treatment suppression without eradication, subsequently leading to relapse after discontinuation of therapy. However, the aspirated vitreous did not reveal *Cladophialophora devriesii*, possibly because the amount of the fungus in the sample was below the detection limits by culturing and molecular methods. After an additional 20 months of antifungal therapy, the infection ultimately resolved. Another attempt was made to discontinue her medication, but again, the eye became inflamed after a period of three months. Vitreous sampling at this stage confirmed the presence of *Cladophialophora devriesii*. Despite initiation of local and systemic antifungal therapy, the patient developed seclusio pupillae with painful secondary glaucoma. Based on an overall assessment and the patient’s preferences, the eye was enucleated almost three and a half years after the first visit. Histopathologic analysis of the enucleated eye showed persistent fungal elements morphologically consistent with *Cladophialophora*. The fungal infection seemed predominantly located within the anterior vitreous, which may explain the nuclear cataract formation, the severe anterior inflammation leading to seclusio pupillae, as well as the relatively high resistance to systemic therapy.

This is the second reported patient with endogenous endophthalmitis caused by *Cladophialophora devriesii*. Our case adds to the limited literature on dematiaceous fungal endophthalmitis and shows how prompt vitreous sampling with microscopy, culturing and gene sequencing contributed to the diagnosis and treatment of this very rare infection. Despite early diagnosis and prolonged local and systemic treatment with modern antifungal agents two recurrences occurred, and it was not possible to achieve long-term control of the fungal infection. In the choice between enucleation and further antifungal treatment with subsequent serious side effects as well as the risk of extraocular spread of the infection, enucleation was considered the best alternative for this patient.

## Data Availability

Additional data that are generated or analysed during this study and not included in the article, are available from the corresponding author on request.

## Abbreviations

BCVA: Best corrected visual acuity
CNS: Central nervous system
COPD: Chronic obstructive pulmonary disease
CT: Computed tomography
IOP: Intraocular pressure
ITS: Internal transcribed spacer
MIC: Minimum inhibitory concentration
PET: Positron emission tomography
16 S rDNA: 16 S ribosomal deoxyribonucleic acid
